# ‘Thrown in at the deep end’: a qualitative analysis into the transition from trainee to consultant during the COVID-19 pandemic and lessons for the future

**DOI:** 10.1016/j.bjao.2023.100217

**Published:** 2023-06-26

**Authors:** Xiaoxi Zhang, Helgi Johannsson, Joseph D. Tucker, Amardeep Riyat, Yuan-Li Tiffany Chiu, Neil Greenberg, Roger Sharpe

**Affiliations:** 1 London North West University Healthcare NHS Trust, London, UK; 2 Imperial College London, London, UK; 3 Imperial College Healthcare NHS Trust, London, UK; 4 London School of Hygiene and Tropical Medicine, London, UK; 5 Department of Medicine, University of North Carolina, Chapel Hill, North Carolina, NC, USA; 6 Centre for Higher Education Research and Scholarship, Imperial College London, London, UK; 7 King's College London, London, UK

**Keywords:** career transition, COVID-19, pandemic, postgraduate medical education, professional development, training, wellbeing, workforce retention

## Abstract

**Background:**

Sustained crises such as the COVID-19 pandemic would be expected to impact the transition from trainee to consultant for anaesthetists or intensivists, but limited research exists on this important topic. This study aimed to examine the social context of this crucial career transition during the pandemic and post-pandemic periods.

**Methods:**

We conducted semi-structured interviews with anaesthetists and intensivists who became consultants after the first UK lockdown. Thematic analysis was used and data saturation was reached at 33 interviews.

**Results:**

The pandemic substantially impacted the transition to consultant role in various ways, including professional identity, clinical and non-clinical responsibilities, and wellbeing. Participants experienced identity confusion, self-doubt, and moral injury, resulting in intense emotional distress, feelings of guilt and helplessness, which persisted beyond the pandemic. They also felt unprepared for their consultant roles because of disruptions in training. The pandemic exaggerated the vulnerability of those transitioning to consultants, because of increased clinical uncertainties, and pressures of the growing backlog. Additionally, the pandemic impacted on the wellbeing of those transitioning to consultants, intensifying feelings of anxiety and stress. We also identified unique opportunities presented by the pandemic, which accelerated learning and encouraged post-traumatic growth. Our study identified practical solutions that may improve transition experience at individual, organisational, and national levels.

**Conclusions:**

Persistent crises significantly impact the transition from trainee to consultant. Our findings generated insights into the challenges of this critical career transition and staff wellbeing, and serve to inform approaches of ongoing support for those transitioning to consultants.

The transition from trainee to consultant is a stressful and vulnerable time in a doctor's career.^1^ The coronavirus disease 2019 (COVID-19) pandemic added challenges to this already demanding transition. Before COVID-19, new consultants often felt that their specialty training prepared them well for their clinical role.^1^,^2^ However, the delivery of anaesthetic and intensive care training in the UK was significantly affected by the pandemic.^3^, ^4^, ^5^ National surgical activity decreased to less than half at the peak of the pandemic with elective surgery especially reduced.^6^ This resulted in fewer opportunities for solo lists, reduced caseload, and decreased case mix for trainees.^7^^,^^8^ Staff sickness, self-isolation, and cancellation of fellowships further limited subspecialty training for senior trainees.^3^^,^^7^ In addition, clinical guidelines rapidly evolved during the pandemic, demanding clinicians to frequently update their knowledge.^9^ COVID-19 also necessitated substantial changes in work pattern, the hospital landscape, resource allocation, and team structure. Furthermore, the pandemic placed an unprecedented burden on healthcare professionals' physical and mental health.^10^^,^^11^ There is lack of research investigating how these important factors influenced the critical career transition from trainee to consultant. As we emerge from the COVID-19 crisis, staff shortages, burnout, and early retirement become the biggest obstacles to tackling the elective backlog and sustaining staff morale.^12^ It is vital to reflect on lessons learnt from the pandemic for the future.

Using qualitative methods, our study explores anaesthetists' and intensivists' experiences of transitioning to the consultant role in the COVID-19 and post COVID-19 period. We also sought to gauge potentially useful sources of support during this important transition, which may help to inform approaches of ongoing support for individuals transitioning to consultants.

## Methods

This study was granted Health Research Authority (HRA) approval on 7 April 2022. Written informed consent was obtained from all participants.

Our methodology was underpinned by the constructivist view that meaning is constructed by people on the basis of their own experiences.^13^ We conducted semi-structured interviews with UK anaesthetists and intensivists who commenced their first consultant job after 23 March 2020, which was the start of the first lockdown in the UK.^14^

We chose qualitative methods because they best capture some of the nuance and tensions related to career transitions. Additionally, there are no validated scales that capture key aspects of career transitions, making a quantitative study difficult. Furthermore, this allowed us to gain a better understanding of career transition in the social context.

A topic guide for the semi-structured interviews was developed by XZ, RS, AR and HJ through an iterative process (see Appendix). This involved literature review, brainstorming key topics, selecting and grouping relevant questions, piloting the topic guide, and refining the questions. Pilot interviews (*n*=3) informed the interview guide but were not included in analysis.

Participants were recruited into the study using three approaches: (1) email to lead consultants and educational supervisors for dissemination among newly appointed consultants within departments; (2) social media (Twitter) calling for potential participants from study investigators; and (3) study advert sent to new consultant social media groups. We verified participant eligibility through the General Medical Council specialist register and the list of consultants achieving a Certificate of Completion of Training (CCT) published in the Royal College of Anaesthetists' Bulletin. Recruitment and interviews were conducted from June to August 2022.

All interviews were video interviews conducted on Microsoft Teams by XZ, who has had training and experience in qualitative research methods. The interviews were transcribed with the Teams utility, but not recorded to ensure a deep, honest discussion. Transcripts were checked and corrected immediately after the interviews, and anonymised.

Iterative analysis was conducted alongside interviews to ascertain the point where data saturation was reached, where no new themes were identified.^13^ After this point, we stopped recruitment. Thematic analysis was used to analyse the interview data.^15^^,^^16^ Analysis was primarily done by XZ using the qualitative data management software NVivo (Version 1.0, QSR International, Massachusetts, USA) with an inductive approach.^15^ The researcher first familiarised themselves with the data by repeatedly reviewing the transcripts while making notes of preliminary ideas. Initial codes were generated from key features of the data in a systematic way. These codes were then organised into broader themes. To ensure accuracy and consistency, codes and themes were independently checked by RS and AR, both of whom have experience in qualitative research. We discussed and debated any differences on the application of codes until a consensus was reached. Finally, the research team examined the thematic map critically to refine the names and meaning for the themes. We carefully considered the prevalence and strength of expression for each theme relating to our research aims within and across the interview transcripts. The methodology was also reviewed externally by JDT and YLTC, both of whom have extensive qualitative research experience.

## Results

We interviewed 33 participants. Interviews lasted an average of 56 min. Characteristics of the participants are summarised in Table 1. Participants' responses did not suggest any patterns based on their gender, age, or region.

**Table 1:** Summary of participant characteristics. CCT, Certificate of Completion of Training.

Characteristics	*N*=33
Gender, *n* (%)	
Female	16 (48.5)
Male	17 (51.5)
Region, *n* (%)	
East of England	2 (6.1)
London	20 (60.6)
Midlands	4 (12.1)
Scotland	1 (3)
South East	3 (9.1)
South West	1 (3)
Wales	2 (6.1)
Age (yr), *n* (%)	
<35	3 (9.1)
35–39	22 (66.7)
40–45	8 (24.2)
>45	0 (0)
Number of months as a consultant, *n* (%)	
<6	4 (12.1)
6–12	11 (33.3)
12–24	13 (39.4)
>24	5 (15.2)
Specialty, *n* (%)	
Anaesthesia	26 (78.8)
Anaesthesia and critical care dual CCT	6 (18.2)
Critical care	1 (3)
Completed a fellowship or advanced module during the COVID-19 pandemic, *n* (%)	
Yes	12 (36.4)
No	21 (63.6)
Less than full time, *n* (%)	
Yes	6 (18.2)
No	27 (81.8)
Academic trainee before CCT, *n* (%)	
Yes	3 (9.1)
No	30 (90.9)

Major themes focused on identity transition, clinical and non-clinical challenges, opportunities in adversity, and wellbeing and support.

### Identity transition

Almost all participants described substantial disruption to work structure. The roles of a consultant became poorly defined with blurred boundaries and varying expectations to support critical care services. This resulted in profound identify confusion.

*‘It wasn’t anywhere near the same as being a normal consultant … It felt like someone put ‘Consultant’ on my badge. It was very fluid, identity wise … I wasn’t really a consultant in any meaningful way.’*

Many participants described a protracted period of uncertainty as they transitioned to be a consultant because of delays in agreeing a job plan and frequent work pattern changes. Some expressed disappointment that the reality of being a consultant deviated wildly from expectation, leading to self-doubt and identity crisis. Several participants questioned their career choice.

*‘You’re building up to this point (CCT) where everything is going to be OK … You’re at the last hurdle … But the entire thing gets smashed to pieces. It makes you question your entire life.’*

Lack of evidence-based guidance at the start of the pandemic, COVID restrictions, and saturated resources led to significant moral injury, wounded identity, and diminished confidence.

*‘I couldn’t provide the level of care I thought I should. It will take a long time to come to terms with … I think back to the things I did during the pandemic … I had my identity that I wanted to build, standards for myself. That’s all gone. I had to bend my ideals.’*

Participants felt that the guilt and moral injury associated with delivering what they considered ‘substandard’ care extended well beyond the pandemic as scarce resources and staff shortages persisted. Some expressed uncertainty for the future and feeling helpless in a failing system.

### Clinical challenges

Most participants who completed advanced modules during the pandemic felt their training was compromised, which led to self-perceived unpreparedness for the consultant role. There was a lack of opportunity for trainees to develop independence because of cancellation of elective surgery. In some cases, the feeling of unpreparedness led to significant anxiety and insecurity.

*‘My fellowship should have allowed me to hone my skills, but it didn’t give me the depth that I expected … When I started my job, I thought, I'm not ready! I barely gave an anaesthetic for over 6 months!’*

In addition, guidelines on COVID management and testing processes evolved rapidly. Most of those interviewed reported that this exacerbated their stress during transition to consultant and caused considerable cognitive overload. The majority of participants commented that clinical decision-making was complicated by COVID and they had little time for reflection because of increased workload.

*‘You stress about your decisions as a new consultant. The pandemic made it worse. Straight forward cases became complicated … I had no time to think about long-term plans. It was just survival, getting through the next few hours.’*

Most participants gave examples of challenging clinical situations. The psychological impact of accepting ultimate responsibility for patients as new consultants was intensified by COVID-19 because of a relatively more morbid patient cohort.

*‘A lot of patients lingering around from the pandemic suddenly became much sicker, needing urgent surgery. There were moments where I thought, I'm scared to be their consultant because there’s no time to optimise them. It felt gruesome and steely.’*

The prolonged demand to support ICU led to many participants feeling deskilled when elective surgery resumed. Some recalled a difficult ‘second transition back to being a normal consultant’ in the post-COVID era.

### Non-clinical challenges

Most participants who transitioned to be consultants during the pandemic described challenges in leadership roles because of changes in team structure and hospital landscape. Some commented that while trying to adjust to their new roles as consultants, working with redeployed staff unfamiliar with ICU caused anxiety.

In contrast, participants who became consultants in the post-pandemic period focused more on difficulties associated with leading an exhausted workforce with low team morale and managing staff shortages. Nearly all participants felt the brief improvement in teamworking at the start of the pandemic rapidly dissipated and continued to deteriorate into the post-COVID period as a result of burnout and increased pressure to reduce the waitlist.

*‘We were far better at collegiate working before the pandemic … Instead of a period of reflection, we’re constantly told our waitlists are too long …It's relentless.’*

Additionally, transition from trainee to supervisor was difficult for some participants because they had to adjust their expectations of trainees who lost training time as a result of redeployment. Several participants commented on an increased demand to provide psychological support to trainees.

Furthermore, most participants struggled with continuing personal and professional development during the pandemic because of cancellation of educational events and overwhelming clinical workload. For a minority of participants, this directly affected applying for consultant jobs and appraisals.

### Opportunities in adversity

This theme was expressed by a minority of participants, but provides a balanced account for those who had a positive career transition during the pandemic. Six participants greatly appreciated opportunities presented by COVID-19, including participation in national research studies, leading quality improvement projects, and making a positive contribution to guidelines. Few participants suggested that COVID-19 accelerated their learning curve as new consultants. One felt strongly that they became more clinically able because of the exposure to a large volume of unwell patients during the pandemic. Participants valued the shared learning with the multidisciplinary team (MDT) and improved management skills through working with redeployed staff. Furthermore, participants appreciated the rapid advancement in health and communication technology, which improved efficiency and productivity.

### Wellbeing and support

Many participants reported poor mental and physical wellbeing during the transition to consultant, especially in the first year of the pandemic. Many described anxiety, stress, and low mood because of uncertainties regarding progression in training, difficult clinical decisions, and poor work-life balance. Mental saturation and physical exhaustion leading to burnout was commonly expressed. Some participants required formal psychological support to manage work-related mental health conditions.

*‘We are like greyhounds, racing to get to the end. Then suddenly, this shiny prize that you’ve been working towards gets absolutely shattered and it breaks you. It left me very broken … It was a dark, dark time.’*

The majority of participants felt they did not receive adequate local induction, which they considered made the transition process rushed and abrupt. Some felt underappreciated and betrayed by their organisations, citing examples such as hospitals cancelling annual leave and not remunerating overtime. Most participants were unaware of any national guidance or support on the transition to consultant.

All participants emphasised the importance of a good support network in maintaining wellbeing during transition to consultant. Opportunities to act up in consultant roles during training helped to build confidence. Support from senior colleagues, particularly constructive reflection on difficult clinical decisions, also eased the transition process.

Participants offered several suggestions for improving the transition experience. Most stressed the importance of self-care. At the organisational level, participants suggested having locally relevant induction and ‘getting the basics right’ (e.g. ensuring correct pay). Some participants recommended the formalisation of mentorship programmes in which newly appointed consultants are paired with senior consultants to provide structured and consistent support throughout the transition process. In addition, participants suggested creating a handbook of useful information (e.g. appraisals, job planning) for senior trainees and new consultants. Several participants advocated formal national guidance on ensuring training progression and protecting staff wellbeing during subsequent pandemics or other emergency settings.

## Discussion

Our results suggest that sustained crises, such as the COVID-19 pandemic, have a substantial and largely negative impact on the transition from trainee to consultant. Interviewees reported adverse impacts on their professional identity, clinical and non-clinical responsibilities, and overall wellbeing. However, a minority reported positive changes suggestive of post-traumatic growth.^17^ Our study expands existing literature by using qualitative methods to examine the social context of this critical career transition during the pandemic and post-pandemic periods. Our findings have important implications for addressing ongoing challenges in workforce retention and staff wellbeing.

We found that transitioning to a consultant role required an intense renegotiation of one's professional identity, consistent with pre-COVID research.^18^ When professional identity misaligns with the work carried out, additional effort is needed to adapt and make sense of the work.^19^ The COVID-19 pandemic necessitated substantial changes in consultants' work structure, workload, and social context.^20^ Most participants were required to work in unfamiliar roles. This fluid role definition and lack of stability led to significant identity confusion and self-doubt.

Previous research reported moral injury in healthcare professionals during the pandemic.^21^, ^22^, ^23^, ^24^ Moral injury refers to intense emotional distress resulting from experiences that violate one's moral or ethical code,^22^ which can lead to maladaptive coping strategies and mental health problems.^25^ Our analysis aligns with the published literature and strongly indicates that those transitioning to consultants during COVID-19 were vulnerable to experiencing moral injury because they felt unable to provide adequate care. Our data also show that feelings of guilt, shame, and helplessness persist into the post-COVID era because overwhelmed resources and staff shortages continued to pose challenges.

Studies on career transitions suggest that new consultants often feel well prepared for their clinical roles.^1^^,^^2^^,^^26^ However, some participants in our study felt unprepared after disrupted training. This is an important finding because self-perceived unpreparedness is associated with burnout.^26^ Accepting ultimate responsibility for patient care has been found to cause anxiety during transition to consultant.^27^ Our analysis supports this and indicates COVID-19 may have intensified vulnerability during career transition and complicated decision making. This resulted from increased clinical uncertainties and pressures of tackling the growing backlog.

Earlier research suggested improved teamwork during the pandemic.^3^ However, participants in our study reported that initial camaraderie within the MDT deteriorated quickly as a result of burnout, which presented challenges in leading and managing teams. This has important implications for COVID recovery, as staff recruitment, retention, and patient care hinge on team morale and cohesiveness.^12^ Furthermore, the transition from trainee to supervisor can be difficult.^1^ Our study showed participants had to provide additional educational and pastoral support to trainees affected by COVID-19, and this need for support is likely to continue beyond the pandemic.

Prior research demonstrated that personal and professional development stagnated during the pandemic.^3^ Although this was the case for most of our participants, some reported unique opportunities, such as rapid integration of research evidence into clinical practice and quick adoption of technology. These experiences fostered confidence and accelerated learning. This is suggestive of post-traumatic growth, where individuals experience positive psychological transformations as a result of effectively handling a traumatic event.^28^ Our findings have implications for developing targeted strategies to promote and support personal and professional development for those transitioning to consultants.

Our analysis provides strong evidence that COVID-19 had a significant and negative impact on mental wellbeing during transition to consultant. Many participants reported heightened levels of anxiety and stress at work and in their personal lives. Some expressed feeling undervalued and considered alternative careers. This is consistent with existing literature.^11^^,^^29^ Pre-COVID data demonstrated a shortage of anaesthetists.^30^ Recent reports show that a third of anaesthetists stated COVID-19 made them less inclined to continue working in the NHS and that one in four consultants planned to leave within five years.^12^^,^^31^ Not feeling valued or supported is one of the main reasons why anaesthetists leave the workforce.^31^

Looking beyond the pandemic, participants in our study proposed a number of practical solutions to improve the transition experience. Although the Royal College of Anaesthetists and Association of Anaesthetists have issued guidance on wellbeing,^32^^,^^33^ our participants were either unaware of such guidance or felt that more specific and comprehensive guidance was required to facilitate career transitions. We propose that a multifaceted approach, incorporating support at the individual, organisational, and national levels, is necessary to improve the transition experience for new consultants (see Table 2). These recommendations have important implications for organisational leaders and policymakers in promoting the wellbeing of new consultants, which is essential for maintaining a resilient and sustainable workforce.

**Table 2:** Support strategies for improving the transition experience from trainee to consultant.

Levels of support	Examples
Individual level	
Building confidence	Encourage senior trainees to act up in consultant roles and make decisions at consultant level in a safe environment supervised and mentored by a senior consultant; create opportunities to enhance professional development, such as actively involving new consultants in research and decision-making processes regarding clinical guidelines.
Network of support	Foster supportive relationships with trusted friends and family; engage in reflective practice with peers and senior colleagues.
Work-life balance and self-care	Establish clear boundaries between work and personal time; recognise the signs of burnout and seek professional support when appropriate.
Organisational level	
Locally relevant induction	Ensure proper onboarding and comprehensive orientation to the department; provide essential resources such as ID cards, computer logins, and access to local clinical guidelines; ensure timely and accurate pay.
Mentorship	Implement or strengthen mentorship programmes pairing new consultants with experienced senior consultants familiar with the department and ideally a second mentor who has recently gone through the transition.
Wellbeing initiatives	Encourage employees to establish clear expectations for work hours and responsibilities; provide adequate practical facilities such as lockers, parking, and rest areas.
National level	
Handbooks	Provide a comprehensive and accessible guide on topics such as job planning, information on CCT, appraisals, becoming an educational supervisor, pension, pay, and private practice.
Guidance on training	Develop guidance on protecting training and ensuring training progression for the next public health emergency.
Advocacy	Advocate for staff wellbeing, fair pay, and sustainable pension schemes using national organisations.

This study has several limitations. First, it is a cross-sectional study with no pre-COVID comparator group which limits our ability to make causal inferences. While a longitudinal study design with a comparator group would have been preferable, it was not feasible given the post-COVID timing of data collection. Second, a significant proportion of our sample was from London. However, participants from various regions of the UK were recruited and no regional differences were observed. Since our study participants were all in the UK, the transferability of the findings to other countries may be limited. Future studies incorporating an international cohort may address this limitation. Third, our sample was a convenience sample, although published data on the anaesthetic workforce suggest that it broadly reflects the characteristics of the population studied.^34^ Finally, the sample size of 33 participants may limit generalisability. However, this sample size is appropriate for a qualitative study,^35^ and the richness of the data collected, combined with the achievement of data saturation, mitigates this limitation.

In conclusion, this study highlights the significant impact of a prolonged crisis, in this case COVID-19, on the transition from trainee to consultant. Our study offers practical solutions to improve the transition experience and generates insights relevant to workforce retention and staff wellbeing.

## Author's contributions

Study design: XZ, RS, HJ, AR

Acquisition of data: XZ

Data analysis: XZ, JDT, AR

Data interpretation: XZ, RS, NG

Drafting of manuscript: XZ

Revision of manuscript: all authors

External review of study design: JDT, YLTC

## Declaration of interest

NG runs a psychological health consultancy called March on Stress.

## Appendix

Semi-structured interview topic guide.

How was your transition to consultant during the COVID-19 pandemic?

Were there any challenges? Tell me more about them.

Did the pandemic have any positive influence on your career transition? If so, how?

Did the pandemic affect your training? If so, how did the impact on training affect your transition to consultant?

How did the pandemic affect your capacity as a new consultant to carry out clinical duties?

How did the pandemic affect your capacity to undertake non-clinical roles of a consultant (e.g. leadership, management, teaching, clinical governance, and teamworking)?

What helped you transition to consultant during the COVID-19 pandemic?

What support do you think should be available?

Do you have any other comments?

The following probes were used when appropriate to allow the participant to elaborate, reflect and compare:

Can you give me an example?

Can you expand on that?

How did that make you feel?

Why do you think … ?

Could I clarify what you said about …, did you mean … ?

You mentioned … can we unpack that a bit more?

What would you say to someone currently going through transition to consultant?

How would you do … differently?

How do you think … changed your practice?

If we had another pandemic, what do you think … should be?
